# Genome-Wide Identification and Expression Analysis of the TIR-NBS-LRR Gene Family and Its Response to Fungal Disease in Rose (*Rosa chinensis*)

**DOI:** 10.3390/biology12030426

**Published:** 2023-03-10

**Authors:** Jurong Song, Feng Chen, Bo Lv, Cong Guo, Jie Yang, Li Huang, Jiaqi Guo, Fayun Xiang

**Affiliations:** Industrial Crops Institute, Hubei Academy of Agricultural Sciences, Wuhan 430070, China

**Keywords:** rose, TIR-NBS-LRR, fungal disease, black spot pathogen, resistance gene

## Abstract

**Simple Summary:**

TIR-NBS-LRR (TNL) is a disease resistance gene family that responds to biotic stress in many plants, but the systematic analysis of this gene family and the expression response to biotic stress have rarely been reported in roses. In the present study, 96 intact TNL gene family members were identified by bioinformatics in *Rosa chinensis*, and analyzed from the perspectives of evolutionary relationships, conserved structures, expression regulation, collinear relationships, and expression patterns. Some of the TNL genes responded to hormones and fungal disease: *RcTNL23* demonstrated strong responses to three hormones and three pathogens. In addition, some TNL genes responded significantly to the black spot pathogen that we isolated, and different members may be involved in different stages of disease defense. In conclusion, we found that the TNL gene family is involved in the response to fungal disease and may function as a disease resistance gene in the rose. The present study lays a theoretical foundation for the functional study of TNL genes and the mining of disease resistance gene in roses, which will inform the selection and breeding of disease-resistant varieties.

**Abstract:**

Roses, which are one of the world’s most important ornamental plants, are often damaged by pathogens, resulting in serious economic losses. As a subclass of the disease resistance gene family of plant nucleotide-binding oligomerization domain (NOD)-like receptors, TIR-NBS-LRR (TNL) genes play a vital role in identifying pathogen effectors and activating defense responses. However, a systematic analysis of the TNL gene family is rarely reported in roses. Herein, 96 intact TNL genes were identified in *Rosa chinensis*. Their phylogenies, physicochemical characteristics, gene structures, conserved domains and motifs, promoter cis-elements, microRNA binding sites, and intra- and interspecific collinearity relationships were analyzed. An expression analysis using transcriptome data revealed that *RcTNL* genes were dominantly expressed in leaves. Some *RcTNL* genes responded to gibberellin, jasmonic acid, salicylic acid, *Botrytis cinerea, Podosphaera pannosa,* and *Marssonina rosae* (*M. rosae*); the *RcTNL23* gene responded significantly to three hormones and three pathogens, and exhibited an upregulated expression. Furthermore, the black spot pathogen was identified as *M. rosae*. After inoculating rose leaves, an expression pattern analysis of the *RcTNL* genes suggested that they act during different periods of pathogen infection. The present study lays the foundations for an in-depth investigation of the TNL gene function and the mining of disease resistance genes in roses.

## 1. Introduction

As sessile organisms, plants have evolved complex immune systems in response to the invasion of pathogens from the external environment. These systems comprise two lines of defense. The first, called pattern-triggered immunity (PTI), is the activation of downstream immune responses by pattern recognition receptors (PRRs). PRRs are localized on cell membranes and recognize pathogen-associated molecular patterns [1,2]. In response to PTI, pathogens secrete effectors that inhibit the activity of PRRs and their complexes, interfering with downstream signaling [3]. Plants have also evolved intracellular nucleotide-binding oligomerization domain (NOD)-like receptors (NLRs). NLRs, also known as nucleotide-binding site–leucine-rich repeats (NBS-LRRs), can directly or indirectly identify pathogen effectors and trigger the plant immune response. The second line of defense in plants is called effector-triggered immunity (ETI) [1,4,5,6]. ETI reactions are more intense than PTI reactions, and can cause hypersensitive responses [5].

Approximately 61% of the more than 310 cloned plant disease resistance genes belong to the NLR gene family [7]. Consequently, NLR proteins, which are also known as resistance gene analogs, have been extensively studied in numerous plants to elucidate their effects on pathogens [4,8]. According to the phylogenetic analysis of NLR genes in angiosperms, they can be divided into three subcategories: TIR-NBS-LRR (TNL), CC-NBS-LRR, and RPW8-NBS-LRR. The N-terminus of TNL protein contains a Toll/interleukin-1 receptor (TIR) domain [9], which is mainly distributed in dicots and is missing in monocots [9,10]. TIR domains typically contain different but highly conserved TIR-1, TIR-2, and TIR-3 motifs [10]. The NBS domain generally contains eight conserved motifs: P-loop (phosphate-binding loop), RNBS-A (resistance nucleotide binding site A), RNBS-B, RNBS-C, RNBS-D, kinase 2, GLPL (Gly-Leu-Pro-Leu, also called kinase 3), and MHDV (Met-His-Asp-Val) [10,11,12,13]. In addition, TNL genes may contain integrated domains (IDs) that serve as bait for pathogen effectors, such as the WRKY domain found in the RRS1 (resistance to Ralstonia solanacearum 1) protein in *Arabidopsis thaliana* (*A. thaliana*) [14,15].

Approximately 60 TNL genes are associated with disease resistance in 21 plant species (Table S1). In *Gossypium hirsutum*, *GhDSC1* responds to verticillium wilt (*Verticillium dahliae*) infection by upregulating expression, and silencing the *GhDSC1* gene makes plants more susceptible to verticillium wilt. A single nucleotide polymorphism (SNP) mutation of the GhDSC1 protein P-loop motif removes the defense response to verticillium wilt in susceptible cotton [16,17]. An *RT4-4* gene involved in the cucumber mosaic virus (CMV) resistance response, which interacts with the elicitor CMV 2a to induce a necrotic response, has been identified in the common bean [18]. The overexpression of the *GmKR3* gene may enhance the resistance of soybeans to a variety of viruses, but does not affect the yield and quality of the plants [19]. Genetic analysis and transgenic verification in potato have proven that the *Gro1-4* gene in the *Globodera rostochiensis* (*G. rostochiensis*) resistance locus is resistant to pathotype Ro1 *G. rostochiensis* [20]. The Glomerella leaf spot (GLS, *Colletotrichum fructicola*) resistance locus was mapped in apple, and *MdTNL1* was considered as a candidate gene [21]. Its overexpression can significantly increase apple resistance to GLS, and an SNP on this gene can be used to distinguish resistant and susceptible germplasms [22]. Table S1 summarizes the TNL genes involved in disease resistance.

As one of the most prominent ornamental plants worldwide, roses are known as the “Queen of flowers” and are widely sold as cut flowers (accounting for approximately 30% of the market), garden plants, and potted plants with important economic value and cultural connotations [23]. However, during cultivation, roses are often damaged by black spot and powdery mildew, which affect their normal growth and flowering [24]. Cut roses are often affected by gray mold during storage and transportation [25]. These three diseases are prevalent among roses worldwide and have a significant impact on their ornamental and economic value. Therefore, it is extremely important to identify disease resistance genes in roses [24]. Map-based cloning is a traditional method for identifying the target genes of a trait, but it is limited by the long study periods required and the complex genomes of roses. Consequently, a few resistance genes have been identified using this method [24]. In recent years, with the rapid development of genomics and sequencing technology, genome-wide gene family analyses have been successfully applied to the mining of disease resistance genes in roses. Examples of such genes include *RcbZIP17* of the *bZIP* family [26], *RcWRKY41* of the WRKY family [27], and *RcERF099* of the AP2/ERF family [28]. These genes participate in resistance to gray mold and have been used in virus-induced gene silencing for the preliminary verification of gene function in roses.

The NLR gene family has been identified in many plants, including members of the Rosaceae family [9,29,30,31,32,33,34,35]. Although the TNL gene family has been identified in *Rosa chinensis* (*R. chinensis*) and an evolutionary analysis has been conducted [33], more in-depth research is required. Therefore, in the present study, to determine the function of the TNL gene family in roses, 96 intact TNL genes were identified in *R. chinensis*. The genes were analyzed from the perspectives of evolution, physicochemical properties, protein structure conservation, gene expression regulation, and collinear relationships. Transcriptome data were also used to analyze the expression pattern of *RcTNL* genes and their response to exogenous hormones and three different pathogens. The black spot pathogen in the roses was identified as *Marssonina rosae* (*M. rosae*). The expression pattern of TNL genes in response to *M. rosae* infection was detected using qRT-PCR. Therefore, we performed a comprehensive analysis of the TNL gene family in *R. chinensis*. Our findings will provide a foundation for the functional study of TNL genes and the identification of disease resistance genes in roses.

## 2. Materials and Methods

### 2.1. Identification of TNL Genes in R. chinensis

Possible TNL gene family members in *A. thaliana* were obtained from The Arabidopsis Information Resource (TAIR) (https://www.arabidopsis.org/, last accessed on 13 August 2022), NIBLRRS website (https://niblrrs.ucdavis.edu/data_links.php, last accessed on 17 August 2022), and from Zhang et al. (2016) [31]. The TNL protein sequences of *A. thaliana* were aligned to the ‘Old Blush’ genome [36] to obtain possible homologous TNL genes in *R. chinensis*. The Batch CD-Search tool provided by NCBI was used to select the Pfam database in order to determine the conserved domains of the proteins, and genes containing TIR, NBS, and LRR domains were obtained. In addition, the Pfam-A.hmm file was obtained from the Pfam website (http://pfam.xfam.org/, last accessed on 18 August 2022), and the Simple HMM Search tool in the TBtools v1.106 software was used to search for genes containing the TIR (Pfam ID: PF01582) and NB-ARC (Pfam ID: PF00931) domains, respectively [37]. The Batch CD-Search tool was used to obtain the conserved domains of the proteins, and to screen for genes that contained all three domains. The results of the two identification methods were combined to obtain the possible TNL genes in *R. chinensis.*

### 2.2. Gene Characteristics and Phylogenetic Analysis

Information about the coding sequence (CDS), number of exons, and distribution of the *RcTNL* genes on the chromosomes was obtained from the ‘Old Blush’ genome annotation file [36]. The distribution of *RcTNL* genes on the chromosomes was visualized using the MG2C tool [38]. The molecular weight, isoelectric point, instability index, aliphatic index, and grand average of hydropathicity of each protein were calculated using the ExPASy ProtParam tool (https://web.expasy.org/protparam/, last accessed on 19 August 2022). The subcellular localization of proteins was predicted using CELLO v2.5 (http://cello.life.nctu.edu.tw/, last accessed on 19 August 2022). The protein sequences of *RcTNL* were first aligned using MUSCLE [39], and a phylogenetic tree was constructed using MEGA X. The evolutionary history was inferred using the maximum likelihood method with 1000 bootstrap replicates and the w/freq. model [40].

### 2.3. Analysis of Gene Structures, Promoters, and Conserved Motifs

The general feature format (GFF) file containing the ‘Old Blush’ genome annotation information was used to obtain the structure of the *RcTNL* gene, and was subsequently combined with the genome file to obtain the sequence 2 kb upstream of the gene initiation codon. This sequence was identified as the promoter sequence. The cis- elements were predicted using the PlantCARE database [41]. The protein sequences of *RcTNL* genes were submitted to the MEME Suite v5.5.1 website to obtain their conserved motif information [42]. The results of this analysis were displayed using TBtools v1.106 software [37].

### 2.4. Prediction of microRNA (miRNA) Target Sites on the Genes

Published *Malus domestica* (*M. domestica*) miRNA sequences were selected from the psRNATarget website to predict the possible miRNA binding sites on *RcTNL* mRNA. Schema V2 (released in 2017) was selected as the scoring schema, but others use the default settings [43].

### 2.5. Analysis of Gene Duplication Events and Collinearity

After BLASTP was used for all rose protein sequences and combined with the GFF file of the genome, MCScanX and TBtools v1.106 software were used to analyze and plot the duplication type and collinearity relationships between the *RcTNL* gene family members [37,44]. GFF files and protein sequences of *Rosa rugosa* (*R. rugosa*) and *Rosa wichuraiana* (*R. wichuraiana*) were obtained from the Genome Database for Rosaceae [45], and the collinearity of the *R. chinensis* genome and those of the two species mentioned above were analyzed and displayed using MCScanX and TBtools v1.106 software [37,44]. Based on the CDS and protein sequences of the *RcTNL* gene pairs, their Ka and Ks values were calculated using TBtools v1.106 software [37].

### 2.6. Transcriptome Data Acquisition and Analysis

The transcriptome data pertaining to different rose tissues were obtained from the SRA database (https://www.ncbi.nlm.nih.gov/sra/?term=, last accessed on 13 December 2022). The study ID for *R. chinensis* cv. ’Old Blush’ root, stem, leaf, and prickle data was SRP200448 and that for *R. chinensis* cv. ‘Old Blush’ flower bud and open flower data was SRP115334. The study ID for six hormone treatments of ‘Samantha‘ was SRP186551 [46]. The study ID for the ‘Samantha’ response to *Botrytis cinerea (B. cinerea)* was SRP120271 [46]. The study ID for the transcriptome data related to the ‘Pariser Charme’ response to *M. rosae* and *Podosphaera pannosa (P. pannosa)* was SRP136240 [47]. The fastq files for the transcriptome data mentioned above were analyzed with the kallisto program [48] using the ‘Old Blush’ genome as a reference [36] to obtain the count and transcripts per million (TPM) values of gene expression. DESeq2 was used to perform differential expression analysis on count data for various comparisons [49]. Differentially expressed genes (DEGs) were identified using the absolute value of a log_2_ fold change ≥ 1 and a padj < 0.05. The gene expression heatmaps were generated using TBtools v1.106, and the log_2_TPM values were used [37].

### 2.7. Isolation, Identification, and Inoculation of the Black Spot Pathogen

The black spot pathogen was isolated from infected rose leaves by monosporic isolation. After DNA of the fungal strain was extracted by E.Z.N.A. ^®^ Fungal DNA Kit (Omega Bio-tek Inc., Norcross, GA, USA), internal transcribed spacer (ITS) sequence was amplified with 2 × KeyPo Mas-ter Mix (Vazyme Biotech Co., Ltd., Nanjing, China ) and then sequenced. MEGA X software was used to construct a phylogenetic tree based on the ITS sequence to determine the pathogen species. The isolated and purified fungal strain (DBE24-1) was transferred to a complete medium (1% glucose, 0.1% yeast extract, 0.1% Ca(NO_3_)_2_·4H_2_O, 0.02% KH_2_PO_4_, 0.025% MgSO_4_·7H_2_O, 0.015% NaCl, 0.05% casein enzymatic hydrolysate, and 0.05% casein acid hydrolysate), incubated at 23 °C while agitating at 180 rpm for approximately 15 days, and filtered through sterilized lens paper to obtain the conidia fluid. A hemocytometer was used to calculate the conidia concentration, which was then adjusted to 2 × 10^5^ colony-forming units (CFUs)/mL by adding 0.1% Tween-20 to the solution. The fourth and fifth leaves from the top of each ‘Old Blush’ plant were picked and sterilized with an aqueous solution of 0.06% to 0.09% NaClO for 2 min, then washed twice with sterile water. Each leaf retained the top three leaflets, which were moisturized and stored after inoculation, as described by Zurn et al. (2018) [50]. The conidia fluid was sprayed evenly on the leaf surface. Three biological replicates were set up at each time-point, and each biological replicate was composed of three leaves of a plant. Sterile water containing 0.1% Tween-20 was used as the control to inoculate the same number of leaves. Samples were collected at 0 h, 24 h, 48 h, 6 days, and 12 days after inoculation, respectively.

### 2.8. qRT-PCR Analysis of RcTNL Genes Response to M. rosae

RNA was extracted from the samples collected in Section 2.7 using a Quick RNA Isolation Kit (Beijing Huayueyang Biotechnology Co., Ltd., Beijing, China) and a PrimeScript™ RT reagent Kit with gDNA Eraser (Takara Bio Inc., Kusatsu, Japan) for reverse transcription synthesis of complementary DNA, all according to the manufacturer’s instructions. Nine pathogen-responsive *RcTNL* genes were randomly selected for qRT-PCR analysis. *UBC* and *GAPDH* were used as reference genes, and the primer sequences are presented in Table S14. The fluorescence signals were detected using a CFX384™ Real-Time System (Bio-Rad, Hercules, CA, USA) after the PCR reaction had been configured using ChamQ Universal SYBR qPCR Master Mix (Vazyme Biotech Co., Ltd., Nanjing, China). Three technical replicates were performed for each biological replicate using the method described by Song et al. (2021) [51]. The relative expression of each gene was calculated using the 2^−ΔΔCT^ method [52]. Statistical analysis of the relative expression levels of the various treatments was performed using a *t*-test on GraphPad Prism 8 (GraphPad Software, Inc., San Diego, CA, USA).

## 3. Results

### 3.1. Identification and Phylogenetic Analysis of the TIR-NBS-LRR (TNL) Genes

**Figure 1:**
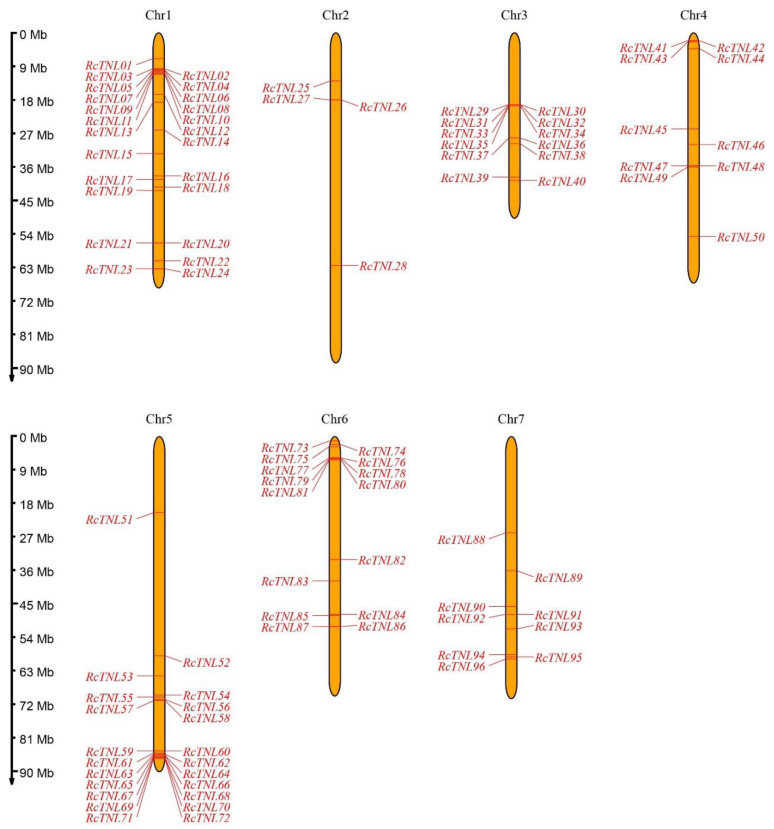
Distribution of TNL genes on *Rosa chinensis* (*R. chinensis*) chromosomes. The rulers on the left indicate the physical size of each chromosome; the ruler unit is megabase (Mb) pair. The black text across the top represents the chromosome number.

A total of 73 TNL genes annotated on the TAIR website, 92 TNL genes from the NIBLRRS website, and 79 TNL genes identified by Zhang et al. (2016) using the Pfam database [31] were combined to obtain 107 TNL genes from *A. thaliana* (Table S2). The sequences of these genes were aligned to the *R. chinensis* reference genome [36] to obtain 1275 possible homologous genes. The conserved domains of these genes were analyzed by the Batch CD-Search tool (NCBI), and 96 of the possible homologous genes contained TIR, NBS, and LRR domains. In addition, using the hidden Markov model (HMM) search method, a total of 183 genes were found to contain both TIR and NBS domains, 95 of which contained TIR, NBS, and LRR domains and were included in the 96 genes. These 96 genes were considered to be the TNL genes of *R. chinensis* and were named *RcTNL01* through *RcTNL96* in order of chromosome number and physical location from low to high (Figure 1, Table S3). They were distributed unevenly across the seven chromosomes of *R. chinensis* with up to 24 on chromosome 1, 22 on chromosome 5, and only 4 on chromosome 2 (Figure 1, Table S3).

**Figure 2:**
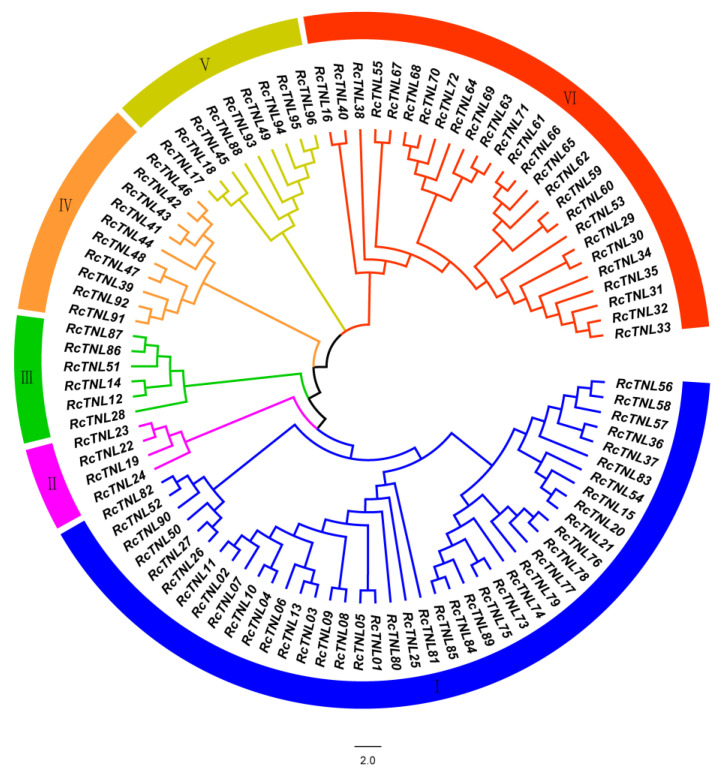
Phylogenetic analysis of TNL proteins in *R. chinensis*. The innermost circle is a phylogenetic tree of *RcTNL* proteins constructed by MEGA X using the maximum likelihood method; the colors represent the six clades. The outermost circle uses blocks of the same six colors to represent the clades; the Roman numerals in the blocks are the clade numbers.

A phylogenetic tree was constructed using the maximum likelihood method to study the evolutionary relationship between *RcTNL* family members in *R. chinensis*. The 96 *RcTNL* genes were divided into six clades, of which, clade I contained the most (up to 41 members) and clade II contained the least (only 4 members) (Figure 2).

### 3.2. Analysis of the Physicochemical Properties of the RcTNL Proteins

The number of exons in the *RcTNL* genes varied greatly from 2 to 19. The RcTNL proteins contained 656 (RcTNL48) to 3431 (RcTNL64) amino acids. The molecular weights of their respective proteins ranged from 74.31 (RcTNL48) to 388.72 kDa (RcTNL64), and the isoelectric points ranged from 5.08 (RcTNL63) to 8.93 (RcTNL95). In total, 87.5% (84) of all of the RcTNL proteins had an instability index greater than 40, indicating that most RcTNL proteins may be unstable. The aliphatic indexes of the RcTNL proteins ranged from 87.38 (RcTNL46) to 106.95 (RcTNL48). The grand average of the hydropathicity of the RcTNL proteins ranged from −0.386 (RcTNL16) to −0.061 (RcTNL77); all of the values were negative, indicating that the RcTNL proteins were hydrophilic. Subcellular localization prediction revealed that 71 (73.96%) RcTNL proteins were in the nucleus, 15 (15.62%) were in the cytoplasm, and 10 (10.42%) were in the plasma membrane (Table S3). This suggests that most RcTNL proteins are primarily intracellular and may be involved in the ETI response.

### 3.3. Domain and Conserved Motif Analysis of the RcTNL Proteins

**Figure 3:**
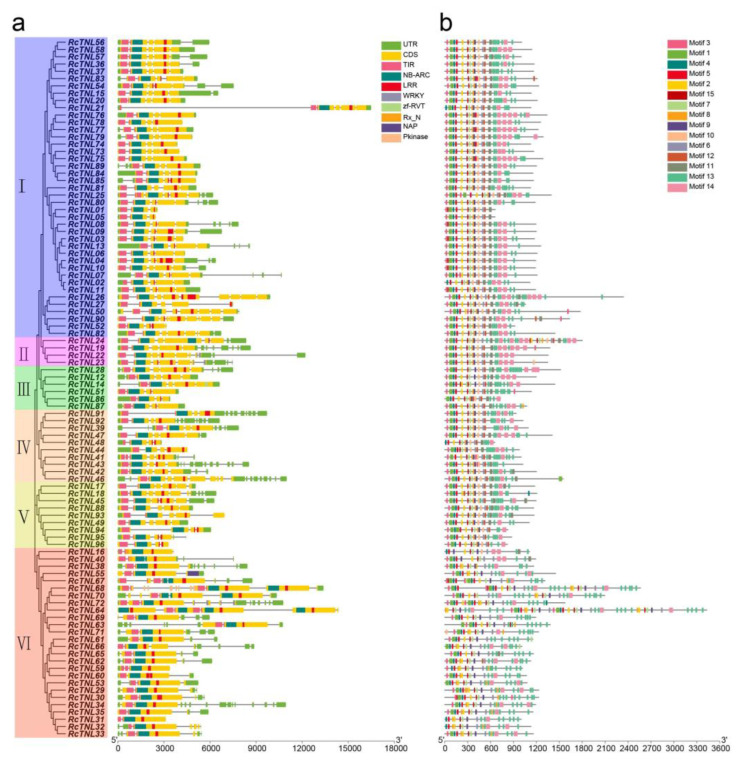
Phylogeny, conserved domains, and motifs of RcTNL proteins and their gene structures. (**a**) Gene structures and conserved domains of the *RcTNL* genes. The phylogenetic tree of the *RcTNL* genes is on the left with the six clades indicated by different background colors. Roman numerals indicate the clade numbers; rectangles of assorted colors to the right represent untranslated regions (UTRs), coding sequences (CDSs), and domains. The horizontal lines connecting rectangles represent introns. The rulers indicate base pair (bp) values. (**b**) Conserved motifs of the RcTNL proteins. The 15 motifs are represented by different colored rectangles. The horizontal lines of tandem rectangles indicate the extent of the protein.

We analyzed the domains of the RcTNL proteins. Their domains were arranged in the order: TIR, NBS, and LRR. We found that 22.92% (22) of the genes contained two to five LRR domains, e.g., RcTNL26 contained five LRR domains. In addition, the WRKY, Rx_N, Pkinase, NAP, and zf-RVT domains were present in 8.33% (8) of the RcTNL proteins (Figure 3a). The additional domains are called IDs. They may act as bait to perceive pathogen effectors, particularly the WRKY, Pkinase, and zf-RVT domains [15,53].

The MEME Suite was used to analyze the conserved motifs of the RcTNL proteins. The motif arrangement was more conserved at the 5′ ends than at the 3′ ends of the proteins. Fifteen motifs were predicted on the RcTNL proteins, and they existed simultaneously on 52.08% (50) of all RcTNL proteins. The probability of each motif appearing on an RcTNL protein was 69.79–100% (Figure 3b, Table S4). Among the identified motifs, TIR-1 (motif 3), TIR-2 (motif 1), TIR-3 (motif 4), TIR-4 (motif 5), RNBS-B (motif 7), RNBS-D (motif 10), GLPL (motif 9), P-loop (motif 2), Kinase 2 (motif 15), and MHDV (motif 6) were also conserved in other species [10,11,13]. TIR-4 (motif 5), MHDV (motif 6), RNBS-B (motif 7), and GLPL (motif 9) were present in all of the RcTNL proteins (Figure 3b), indicating that these four motifs might have crucial functions.

### 3.4. Cis-Element Analysis of the RcTNL Promoters

**Figure 4:**
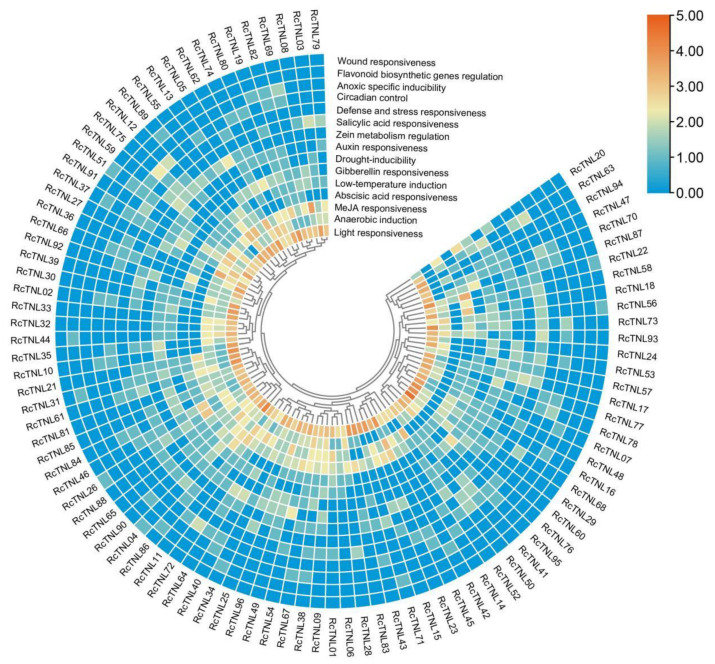
Classification and number of cis-elements in the promoter regions of the *RcTNL* genes. The heatmap was plotted by taking log_2_ fold of the number of cis-elements. The color shift from blue to red indicates an increasing number of cis-elements.

Transcription factors can regulate the expression of genes by binding cis-elements in the promoter region. The PlantCARE database was used to predict the promoter region cis-elements that were used to study the regulation of *RcTNL* gene expression. The light responsive elements in the promoter region of each *RcTNL* gene were most abundant and were present in all of the genes; each gene contained at least two. The *RcTNL* gene promoter regions contained cis-elements associated with jasmonic acid (JA), abscisic acid (ABA), gibberellin (GA), auxin, and salicylic acid (SA), indicating that the expression of *RcTNL* genes may be regulated by these five hormones. Cis-elements related to stress and defense were also present in the promoter regions of the *RcTNL* genes (Figure 4 and Figure S1, Table S5), indicating that *RcTNL* genes may be involved in the defense against biotic and abiotic stress, as reported in other species [12,14,54].

### 3.5. Prediction of Target Binding Sites for miRNAs on the RcTNL Genes

**Figure 5:**
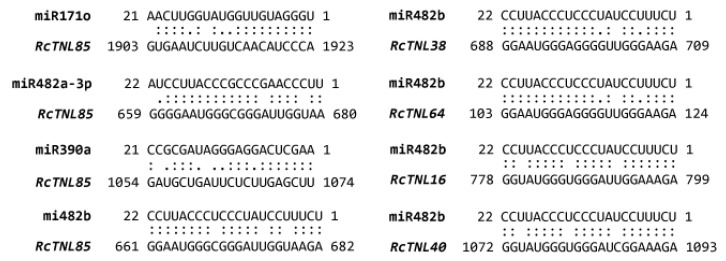
Possible microRNA binding sites for five *RcTNL* genes. Double dots between bases indicate a successful pairing, single dot indicates an additional pairing between U and G, and a blank indicates no pairing.

In plants such as *A. thaliana* [54], soybean [55], and tomato [56], miRNAs can regulate plant immune responses to pathogens by targeting TNL genes. To determine whether *RcTNL* genes were targeted by miRNAs, 207 published *M. domestica* miRNA sequences were selected from the psRNATarget website to predict possible miRNA binding sites on the *RcTNL* genes [43]. A total of 164 miRNAs from 42 miRNA families were identified. These miRNAs regulate genes primarily at the transcription level in a cleavage manner (Table S6). Among them, the miR482 family targeted the most *RcTNL* genes at 67 (69.79%), followed by the miR171, miR169, and miR396 families, which targeted 26 (27.08%), 20 (20.83%), and 17 (17.70%) *RcTNL* genes, respectively. Moreover, we found that multiple miRNAs were capable of targeting a single *RcTNL,* e.g., *RcTNL85* might be regulated by 13 miRNAs. One miRNA was also capable of targeting multiple *RcTNL* genes, e.g., miR482b could target 61 *RcTNL* genes (Figure 5, Table S6). This is similar to previous studies [13,57].

### 3.6. Duplication and Collinearity Analysis of the RcTNL Genes

A collinearity analysis of the *R. chinensis* genome revealed four pairs of *RcTNL* genes with segmental duplication events (including eight *RcTNL* genes) and two pairs of *RcTNL* genes with tandem duplication events located on chromosomes 3 and 5 (Table 1, Figure S2). The *RcTNL* gene pairs were in the same evolutionary clade and were mainly located in clades I and VI. In addition, 12 *RcTNL* genes had a collinearity relationship with 14 non-TNL genes, of which, *RcTNL04* and *RcTNL42* had segmental duplication events with two non-TNL genes, respectively (Table S7).

To study the selection pressure of the *RcTNL* genes at the protein level, the Ka to Ks ratio of the *RcTNL* gene pairs was calculated. The Ka/Ks values of the six *RcTNL* gene pairs ranged from 0.443 to 0.653. All values were less than 1, indicating that these *RcTNL* gene pairs underwent purification or negative selection (Table 1). This is similar to the observation that NLR genes in Rosaceae also undergo purification selection [33].

**Figure 6:**
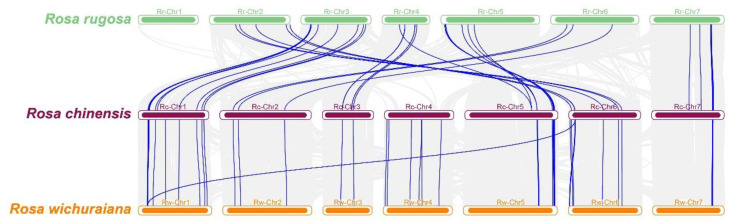
Collinearity of *R. chinensis* with *Rosa rugosa* (*R. rugosa*) and *Rosa wichuraiana* (*R. wichuraiana*). Chromosomes of *R. rugosa*, *R. chinensis*, and *R. wichuraiana* are indicated by green, purple, and orange bars, respectively, and are arranged by chromosome number from low (left) to high (right). The blue lines between the chromosomes of the different species indicate collinearity blocks with *RcTNL* genes, and the gray lines indicate blocks of collinearity between genomes.

A collinearity analysis of *R. chinensis* with *R. rugosa* and *R. wichuraiana* revealed that, whether based only on TNL genes or on all genes, the collinear relationship between *R. chinensis* and *R. wichuraiana* was stronger than that between *R. chinensis* and *R. rugosa*. Of the 96 *RcTNL* genes, 41 were collinear with *R. rugosa* and 45 were collinear with *R. wichuraiana* (Figure 6, Table S8).

### 3.7. Expression Pattern Analysis of the RcTNL Genes

**Figure 7:**
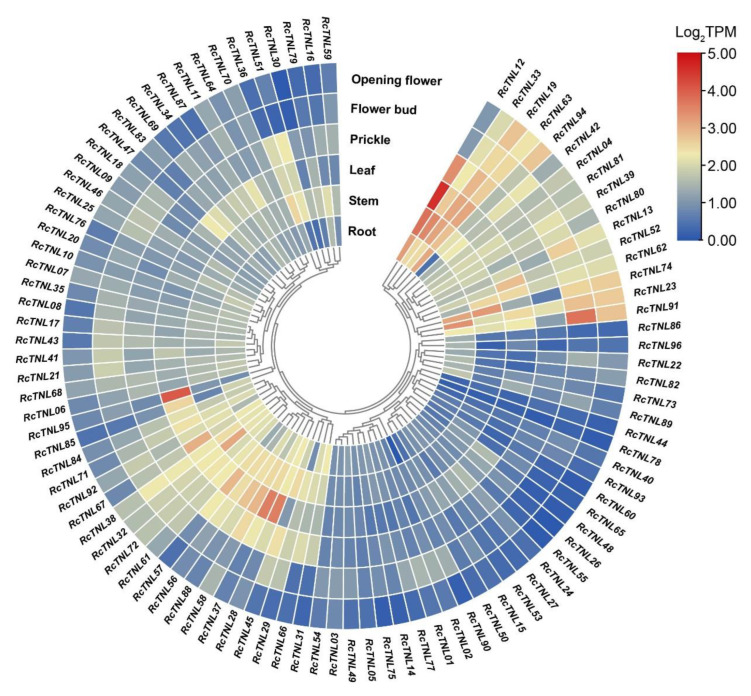
Expression patterns of *RcTNL* genes in various *R. chinensis* tissues. The expression of *RcTNL* genes in the various tissues is represented on a heatmap using log_2_TPM (transcripts per million) values. The innermost circle shows clustering by gene expression patterns. The blocks change from blue to red, indicating a change in the log_2_TPM values from low to high.

We analyzed the ‘Old Blush’ transcriptome data from the root, stem, leaf, prickle, flower bud, and open flower published on the SRA database. The expression of *RcTNL* genes in the leaf was higher than in any of the other tissues, followed by the stem and root. Among these genes, *RcTNL12*, *RcTNL95*, and *RcTNL28* exhibited higher expression levels in the leaf, and *RcTNL12*, *RcTNL33*, and *RcTNL74* exhibited higher expression levels in the stem. *RcTNL33*, *RcTNL19*, and *RcTNL63* exhibited high expression levels in all tissues. Some *RcTNL* genes were only highly expressed in specific tissues, e.g., *RcTNL95* in leaf (Figure 7, Table S9). These results indicate that, under non-pathogenic conditions, the overall expression of *RcTNL* genes is low, but a few genes exhibit higher expression levels in specific tissues.

### 3.8. Response of the RcTNL Genes to Exogenous Hormones

**Figure 8:**
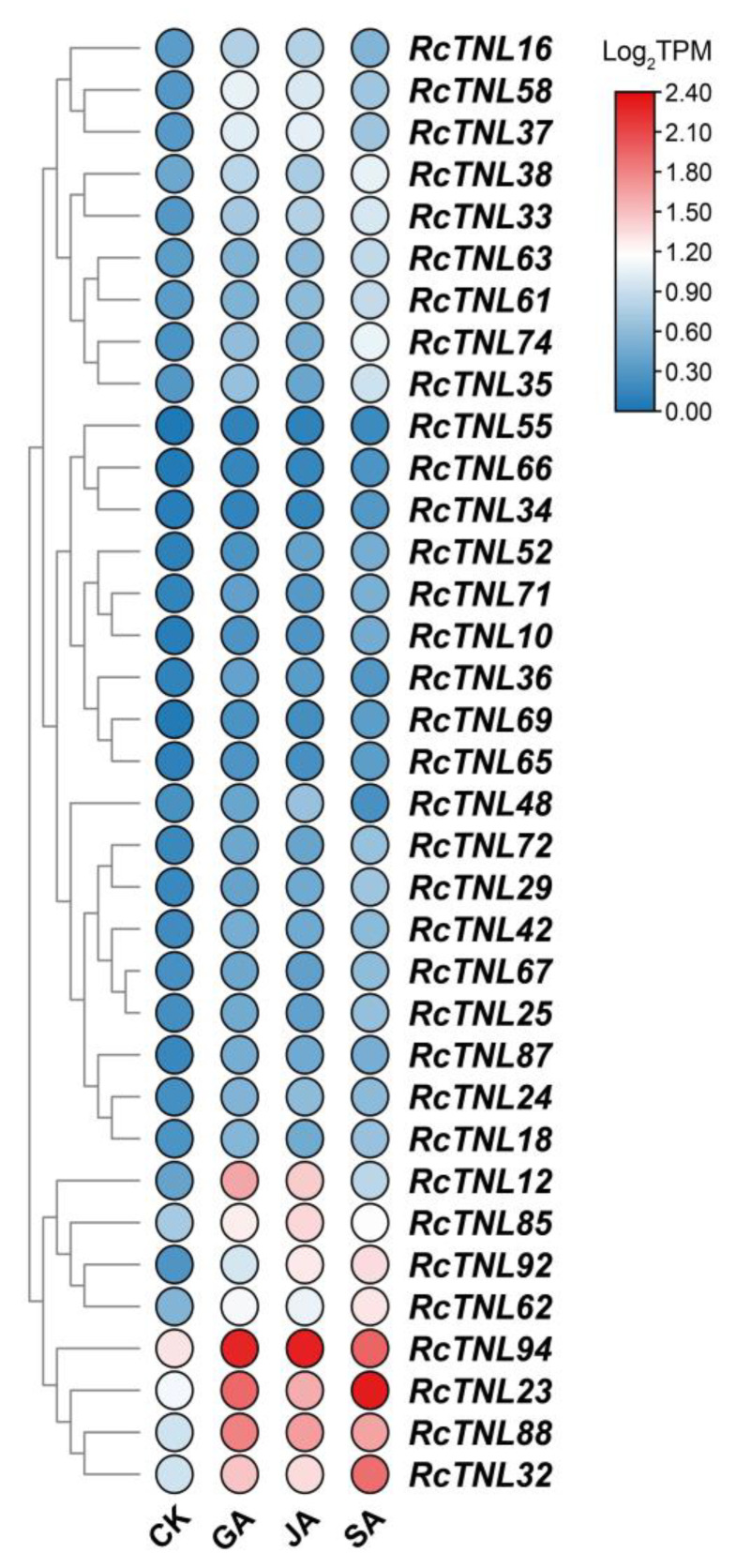
Heatmap of *RcTNL* genes differentially expressed in ‘Samantha’ petals after hormone treatment. CK denotes treatment with deionized water (the control), and GA, JA, and SA denote treatment with gibberellin, jasmonic acid, and salicylic acid, respectively. The change in color of the circles from blue to red indicates the change in the log_2_TPM values from low to high.

Because the promoter regions of some *RcTNL* genes contain hormone responsive elements (Figure 4), it is possible that they are regulated by exogenous hormones. The transcriptome sequencing was performed on petals after treatment with six hormones (GA, JA, SA, ABA, 1-naphthaleneacetic acid (NAA), and 2,4-dichlorophenoxy acetic acid (2,4-D)) and deionized water (CK) using ’Samantha’ cut flowers [58]. The differentially expressed *RcTNL* genes of six hormonal treatments compared to CK were analyzed separately. The results revealed that 15, 9, and 26 *RcTNL* genes were differentially expressed after GA, JA, and SA treatment, respectively, but the remaining hormone treatments did not result in the differential expression of the *RcTNL* genes. Integrating the differential expression of the *RcTNL* genes after the three effective hormone treatments (GA, JA, and SA), 35 *RcTNL* genes were found to be differentially expressed after at least one hormone treatment, and all of them exhibited different degrees of upregulated expression (Figure 8, Table S10). Combining the distribution of the three hormone response elements on the promoters of these *RcTNL* genes revealed that 60%, 44.44%, and 30.77% of the *RcTNL* genes that responded to GA, JA, and SA, respectively, contained corresponding cis-elements on the promoters (Figure 4, Table S5). This indicates that these *RcTNL* genes may be directly or indirectly regulated by these three hormones.

### 3.9. Response of the RcTNL Genes to Fungal Pathogens

**Figure 9:**
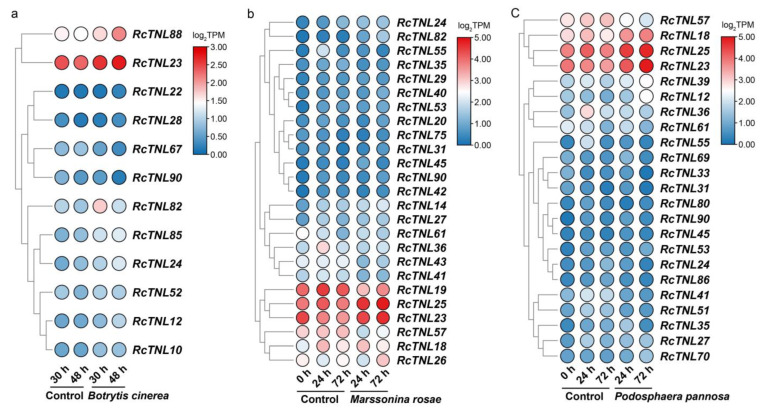
Expression pattern of *RcTNL* genes in response to fungal disease. (**a**) Heatmap of *RcTNL* genes differentially expressed in ‘Samantha’ petals during inoculation with *Botrytis cinerea*. (**b**) Heatmap of *RcTNL* genes differentially expressed in ‘Pariser Charme’ leaves during inoculation with *Marssonina rosae*. (**c**) Heatmap of *RcTNL* genes differentially expressed in ‘Pariser Charme’ leaves during inoculation with *Podosphaera pannosa*. The change in color of the circles from blue to red indicates a change in the log_2_TPM values from low to high.

In order to investigate the response of *RcTNL* genes to fungal pathogens, transcriptome data were analyzed during infection by *B. cinerea*, *M. rosae*, and *P. pannosa* [46,47]. At 30 h and 48 h after the infection of ‘Samantha’ petals by *B. cinerea*, 1 and 11 *RcTNL* genes were differentially expressed, respectively, compared to the expression in the control petals. This indicates that the response of the *RcTNL* genes to *B. cinerea* mainly occurred at 48 h. After inoculation with *B. cinerea*, four *RcTNL* genes were differentially expressed between the two time-points. The differentially expressed *RcTNL* genes in the two comparisons described above were integrated; 12 genes were differentially expressed in at least one comparison and were considered to respond to *B. cinerea*. These genes will be referred to as *RB-TNL* genes. Compared to the uninfected control, 8 and 10 *RB-TNL* genes were upregulated at 30 h and 48 h, respectively. Eight *RB-TNL* genes were upregulated at 48 h after infection rather than at 30 h (Figure 9a, Table S11). It is likely that these differentially expressed members of the *RcTNL* family participate in the defense response against *B. cinerea*, mostly through an increased expression.

In ‘Pariser Charme’ leaves infected with *M. rosae*, 12 and 5 differentially expressed *RcTNL* genes were observed at 24 h and 72 h of infection, respectively, compared to in the control leaves. A total of 14 (24 h vs. 0 h), 6 (72 h vs. 24 h), and 12 (72 h vs. 0 h) differentially expressed *RcTNL* genes were observed between the time-points. After integrating these DEGs, 25 *RcTNL*s were found to be differentially expressed in at least one comparison and were considered to respond to *M. rosae*. These genes will be referred to as *RM-TNL* genes. Compared to the control leaves, 17 *RM-TNL* genes exhibited a downregulated expression at 24 h, and 17 *RM-TNL* genes exhibited an upregulated expression at 72 h of infection. The expression pattern of the *RM-TNL* gene during infection with *M. rosae* was characterized by both continuous upregulation (10 genes) and downregulation followed by upregulation (10 genes; Figure 9b, Table S12).

‘Pariser Charme’ leaves were inoculated with *P. pannosa* and followed through three time-points. Compared to the control leaves, 7 and 12 differentially expressed *RcTNL* genes were observed at 24 h and 72 h of infection, respectively, and 5 (24 h vs. 0 h), 8 (72 h vs. 24 h), and 13 (72 h vs. 0 h) differentially expressed *RcTNL* genes were observed between the time-points. After integrating these DEGs, 23 *RcTNL*s were found to be differentially expressed in at least one comparison in response to *P. pannosa*. These genes will be referred to as *RP-TNL* genes. Compared to the control leaves, 13 *RP-TNL* genes exhibited a downregulated expression at 24 h and 12 *RP-TNL* genes exhibited an upregulated expression at 72 h. The *RP-TNL* genes were mainly upregulated and then downregulated (8 genes), continuously upregulated (7 genes), and continuously downregulated (6 genes) during *P. pannosa* infection (Figure 9c, Table S13).

A total of 15 common genes were differentially expressed in ’Pariser Charme‘ leaves infected with *M. rosae* and *P. pannosa*, of which, seven exhibited the same expression patterns at all three time-points of infection with the two pathogens (Figure 9b,c). It is possible that these genes participate in defense against these two pathogens in similar ways. *RcTNL23*, *RcTNL24,* and *RcTNL90* were differentially expressed in the roses inoculated with the three pathogens; only *RcTNL23* had the same expression pattern during infection with the three pathogens, and all exhibited an upregulated expression. This indicates that *RcTNL23* may participate in defense against the three pathogens in the same way. *RcTNL24* was upregulated during infection with *M. rosae* and *B. cinerea*, and first upregulated and then downregulated during infection with *P. pannosa*. However, the expression pattern of *RcTNL*90 was different during the infection of three pathogens (Figure 9). The results indicate that, when dealing with different types of pathogens, members of the *RcTNL* family may have different functions.

### 3.10. Expression Patterns of the RcTNL Genes in Response to M. rosae

**Figure 10:**
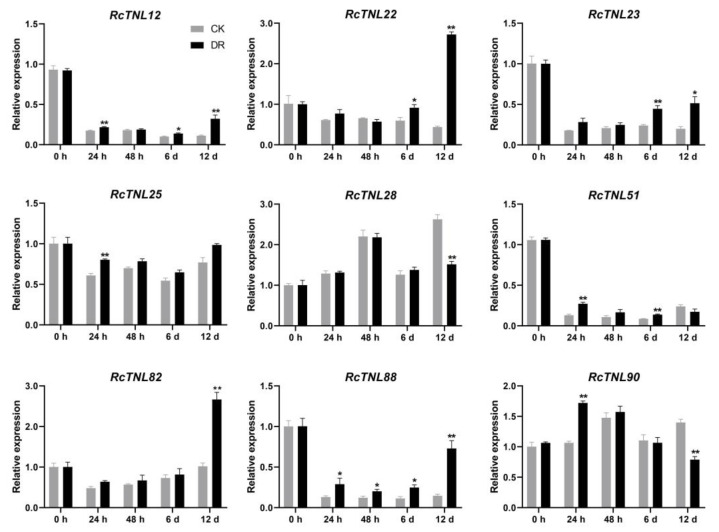
Expression patterns of nine *RcTNL* genes during infection with *Marssonina rosae*. The gray and black columns indicate the relative expression levels (mean ± standard deviation) of the *RcTNL* genes after inoculation with sterile water (CK) and DBE24-1 conidia (DR), respectively. Statistical analysis of the relative expression levels of CK and DR was performed using a *t*-test; * and ** indicate adjusted *p*-values of < 0.05 and < 0.01, respectively.

Rose leaves infected with black spot disease in the field were used for pathogen isolation. A phylogenetic tree was constructed after amplifying the ITS sequence of the isolated strain (DBE24-1), sequencing, and alignment. The pathogen was identified as *M. rosae* (Figure S3), as in a previous study [59,60,61]. The surfaces of leaves from the two groups of ’Old Blush‘ were inoculated with DBE24-1 (DR) and sterile water (CK), respectively, and samples were collected at 0 h, 24 h, 48 h, 6 days, and 12 days post-inoculation. Black spots began to appear on the leaves after 6 days, and at 12 days, the disease index was determined (Figure S4), as in a previous study [59]. The disease index of ‘Old Blush’ was 48.89. Of the *RcTNL* genes identified in response to pathogens (Figure 9), nine were randomly selected for qRT-PCR analysis. Compared to the control, all nine *RcTNL* genes were significantly upregulated at one or more time-point after *M. rosae* inoculation, while *RcTNL88* showed up-regulation at all four time-points after inoculation. The response of these genes to *M. rosae* was the most at 12 days, followed by 6 days and 24 h, whereas only *RcTNL88* responded at 48 h (Figure 10, Table S15).

## 4. Discussion

Roses are important ornamental plants, and are widely planted worldwide. Their growth and flower quality is often damaged by various diseases, including black spot, powdery mildew, and gray mold. However, only a small number of disease resistance genes have been cloned and studied [24,25,47,62]. In recent years, the publication of the reference genome and related omics data pertaining to Rosa plants has provided a shortcut for the identification of disease resistance genes [23,36,45]. As a subclass of the NLR gene family, TNL genes have similar functions in terms of the defense response to pathogens. In plants, the immune response to pathogens is mainly triggered by the direct or indirect identification of pathogen effectors [5,8]. TNL genes have been reported as disease resistance genes in a variety of plants (Table S1), such as cotton [17], soybean [19], apple [22], and potato [20]. In the present study, the TNL gene family was identified in *R. chinensis* and analyzed by bioinformatics. Transcriptome data were used to mine TNL genes that were possibly involved in the defense response, and the responses of nine TNL genes to *M. rosae* were detected by qRT-PCR.

### 4.1. Frequency and Duplication Type of the RcTNL Genes

The frequency of identified TNL genes varies greatly among plant species. For example, the frequency of intact TNL genes is as high as 0.319% in *Vitis vinifera* [29], 0.215% in *Euryale ferox* [34], 0.117% in potato [12], and only 0.003% in *Actinidia chinensis* [32]. In the present study, 96 intact TNL genes containing TIR, NBS, and LRR domains were identified in *R. chinensis*, and their frequency was 0.211%, similar to that in *Euryale ferox*. Guo et al. (2022) reported a frequency of intact TNL genes of only 0.01% in *R. chinensis* [33]. This difference may have been due to the different genomes and identification methods used for TNL genes.

A collinearity analysis of the genome revealed that a total of 18 segment and 13 tandem duplication events contributed to the expansion of the TNL gene family in *R. chinensis*, and occurred mainly between TNL and non-TNL genes (Table S7). However, there were four segment and two tandem duplication events between the TNL genes. This corroborates the results reported by Dubey et al. (2022) following their analysis of TNL gene duplication events in potato [12]. We believe that this was mainly due to the intact TNL genes identified in the present study. Genes that did not simultaneously comprise three domains were non-TNL genes, even if they contained two domains. This may explain why some TNL genes have a collinear relationship with non-TNL genes.

### 4.2. Possible Functions of RcTNL Protein Domains

NLR proteins usually contain TIR, CC, RPW8, NBS, and LRR domains. TNL proteins, which constitute a subclass of NLR proteins, mainly contain TIR, NBS, and LRR domains. The TIR domain can recognize and interact with pathogen effectors, and its heterodimization is required for the formation of functional immune receptor complexes [63]. NBS domains can bind and hydrolyze ATP, transform proteins from active to inactive states, and act as molecular switches in the signal transduction process [64,65]. The LRR domain affects the specificity of NLR proteins for effector recognition [66], and mediates the interaction of NLR proteins with pathogen effectors [67]. In the present study, 96 RcTNL proteins contained these three domains, of which, 22.92% contained two to five LRR domains. These conserved domains may be critical for the immune response in roses.

TNL proteins contain other IDs in addition to the three domains described above. Among these, the WRKY and kinase domains are the most common, and may be used as bait for pathogen effectors to participate in plant defense [15,53]. In the present study, the RcTNL proteins contained five IDs distributed on eight RcTNL proteins. Of these, three RcTNL proteins contained the WRKY domain (Figure 3a). In *A. thaliana*, the WRKY domain of RRS1 (a TNL protein) is capable of detecting pathogen effectors that target the WRKY protein, which is involved in pathogenic defense [14]. In the present study, the response of *RcTNL* genes to three pathogens was analyzed (Figure 9). Six out of eight *RcTNL* genes containing IDs were involved in the pathogen response. Among these genes, *RcTNL23*, which contains a WRKY domain, responded strongly to all of the pathogens tested. It may have played an important role in the defense against the three fungal diseases. *RcTNL55*, which contains an NAP domain, responded to both *M. rosae* and *P. pannosa*, and may be involved in the defense against these two pathogens. In *A. thaliana*, the domains of interaction between NLR proteins and pathogen effectors contain various IDs, including WRKY and NAP [53]. Therefore, it is speculated that RcTNL proteins may also bind to pathogen effectors by their IDs, thereby participating in defense against pathogens. Gene silencing or gene editing techniques can be used to further validate the function of *RcTNL* genes.

### 4.3. Regulation of RcTNL Gene Expression

An analysis of the promoter region of the *RcTNL* gene revealed the presence of cis-elements related to plant hormones and the defense response (Figure 4, Table S5). Therefore, the transcriptome data pertaining to cut rose flowers treated with six hormones (2,4-D, ABA, GA, JA, NAA, and SA) were analyzed. Compared to the control, 15, 9, and 26 *RcTNL* genes were upregulated to varying degrees after GA, JA, and SA treatment, respectively, and responded to the hormones. As in plants such as cotton [17], cassava [35], and grape [68], we found that the TNL genes were significantly upregulated after the exogenous application of SA. In addition, the TNL genes involved in disease resistance in cassava [35] and grape [68] exhibited similar expression patterns during exogenous SA application and pathogen infection.

In the present study, the cis-elements related to defense and stress responsiveness on the *RcTNL* promoters were mainly TC-rich repeats (Table S5), which are also present in the promoter region of the *VpTNL1* gene in *Vitis pseudoreticulata*. The promoter truncation experiment further suggested that this element may play an important role in the immune response to *Erysiphe necator*. In addition, TCA elements in the promoter region of this gene are associated with the response to SA [68]. In the present study, the SA response elements in the promoter region of the *RcTNL* genes were also mainly TCA elements (Table S5), and it is speculated that this element is also important for the SA regulation of *RcTNL* expression.

miRNA is involved in the expression regulation of genes as a negative regulator, and many studies have shown that it regulates the defense response of plants by targeting NLR genes [31]. The miR482 family, which reportedly targets the NLR gene family in various plants such as tomato [69], potato [70], and cotton [71], is involved in the defense response to pathogens. In the present study, we predicted possible miRNA binding sites on the *RcTNL* genes and found that the miR482 family targeted 69.79% of the *RcTNL* genes. Therefore, the miR482 family may be the main candidate for research into the regulation of *RcTNL* gene expression. The expression patterns of miRNAs and their targeted *RcTNL* genes in response to pathogens, combined with transgene, transcriptome, and degradation sequencing technologies, may provide further verification. It has been reported that miRNAs mainly target conserved regions of NLR genes, such as P-loop, Kinase-2, and MHDV motifs in the NB-ARC domain [31,72]. This may have been the reason for why a single miRNA was capable of targeting multiple *RcTNL* genes in the present study.

## 5. Conclusions

In the present study, 96 intact TNL family genes were identified in *R. chinensis* by bioinformatics. A phylogenetic analysis enabled us to divide the genes into six clades. It was predicted that RcTNL proteins would be mainly localized in the nucleus and would be unstable and hydrophilic. Eight of the RcTNL proteins contained IDs in addition to TIR, NBS, and LRR domains. Multiple conserved RcTNL protein motifs also existed in multiple species. Most of the *RcTNL* gene promoter regions contained cis-elements associated with hormones, stress, and defense, and there were miRNA target sites on the *RcTNL* genes. Segmental and tandem duplication events in the *RcTNL* genes may have contributed to the expansion of this gene family.

Compared to the control, 15, 9, and 26 *RcTNL* genes were upregulated and differentially expressed in cut rose petals after treatment with GA, JA, and SA, respectively. In addition, 12, 25, and 23 *RcTNL* genes responded to *B. cinerea*, *M. rosae*, and *P. pannosa*, respectively. Among these, *RcTNL23*, *RcTNL24*, and *RcTNL90* all responded to the three pathogens, and *RcTNL23* exhibited an upregulated expression. We isolated and identified the rose black spot pathogen as *M. rosae*, and inoculated rose leaves with it. The expression patterns of nine *RcTNL* genes in five stages of infection with *M. rosae* were obtained by qRT-PCR analysis, and the responses of the *RcTNL* genes to the pathogens were further verified. The results of the present study will lay the foundation for the functional study of TNL genes in roses, and may inform the mining of disease resistance genes and the selection and breeding of resistant rose varieties.

## Figures and Tables

**Table 1:** Ka/Ks ratio of the duplicated *RcTNL* gene pairs in collinear blocks.

Duplicated Gene Pairs		Clades	Ka	Ks	Ka/Ks	Duplication Type
*RcTNL10*	*RcTNL81*	I	0.346	0.798	0.433	Segmental
*RcTNL63*	*RcTNL69*	VI	0.253	0.387	0.653	Segmental
*RcTNL07*	*RcTNL11*	I	0.062	0.101	0.611	Segmental
*RcTNL64*	*RcTNL72*	VI	0.134	0.264	0.508	Segmental
*RcTNL34*	*RcTNL35*	VI	0.116	0.187	0.623	Tandem
*RcTNL65*	*RcTNL66*	VI	0.198	0.398	0.496	Tandem

## Data Availability

All data generated or analyzed during this study are included in this published article and its Supplementary Information Files.

## Supplementary Materials

The following supporting information can be downloaded at: https://www.mdpi.com/article/10.3390/biology12030426/s1, Figure S1: Distribution of cis-elements on the *RcTNL* gene promoters; Figure S2: Segmental duplicated *RcTNL* gene pairs in *R. chinensis*; Figure S3: Molecular identification of the black spot pathogen strain DBE24-1 in the rose; Figure S4: Black spot symptoms on ‘Old Blush’ leaves after inoculation with the DBE24-1 strain; Table S1: Information on TNL genes as resistance genes; Table S2: Collection and combination of TNL genes in Arabidopsis; Table S3: Characteristics of TNL genes in *R. chinensis*; Table S4: Conserved motifs information of TNL proteins in *R. chinensis*; Table S5: Cis-elements information of TNL gene promoters in *R. chinensis*; Table S6: Predicted binding sites on TNL genes targeted by miRNA in *R. chinensis*; Table S7: Information on TNL gene duplication events in *R. chinensis*; Table S8: Orthologous TNL gene pairs between *R. chinensis* and two other species; Table S9: TPM values of TNL genes in different tissues of *R. chinensis*; Table S10: TPM values of TNL genes differentially expressed in response to hormones in *R. chinensis*; Table S11: TPM values of TNL genes differentially expressed in response to *Botrytis cinerea* in *R. chinensis;* Table S12: TPM values of TNL genes differentially expressed in response to *Marssonina rosae* in *R. chinensis*; Table S13: TPM values of TNL genes differentially expressed in response to *Podosphaera pannosa* in *R. chinensis*; Table S14: Sequence information of primers. Table S15. qRT-PCR results of nine *RcTNL* genes after inoculation with *Marssonina rosae.*
