# Supplementary material for: Genome-Wide Identification and Expression Analysis of the TIR-NBS-LRR Gene Family and Its Response to Fungal Disease in Rose (Rosa chinensis)
Source: Biology (Basel). 2023 Mar 10;12(3):426. doi: 10.3390/biology12030426 (PMC10045381; doi:10.3390/biology12030426)
Supplement: Supplementary file 1 [file biology-12-00426-s001.zip › biology-2213163_Supplementary Figures.pdf]

Supplementary Figures

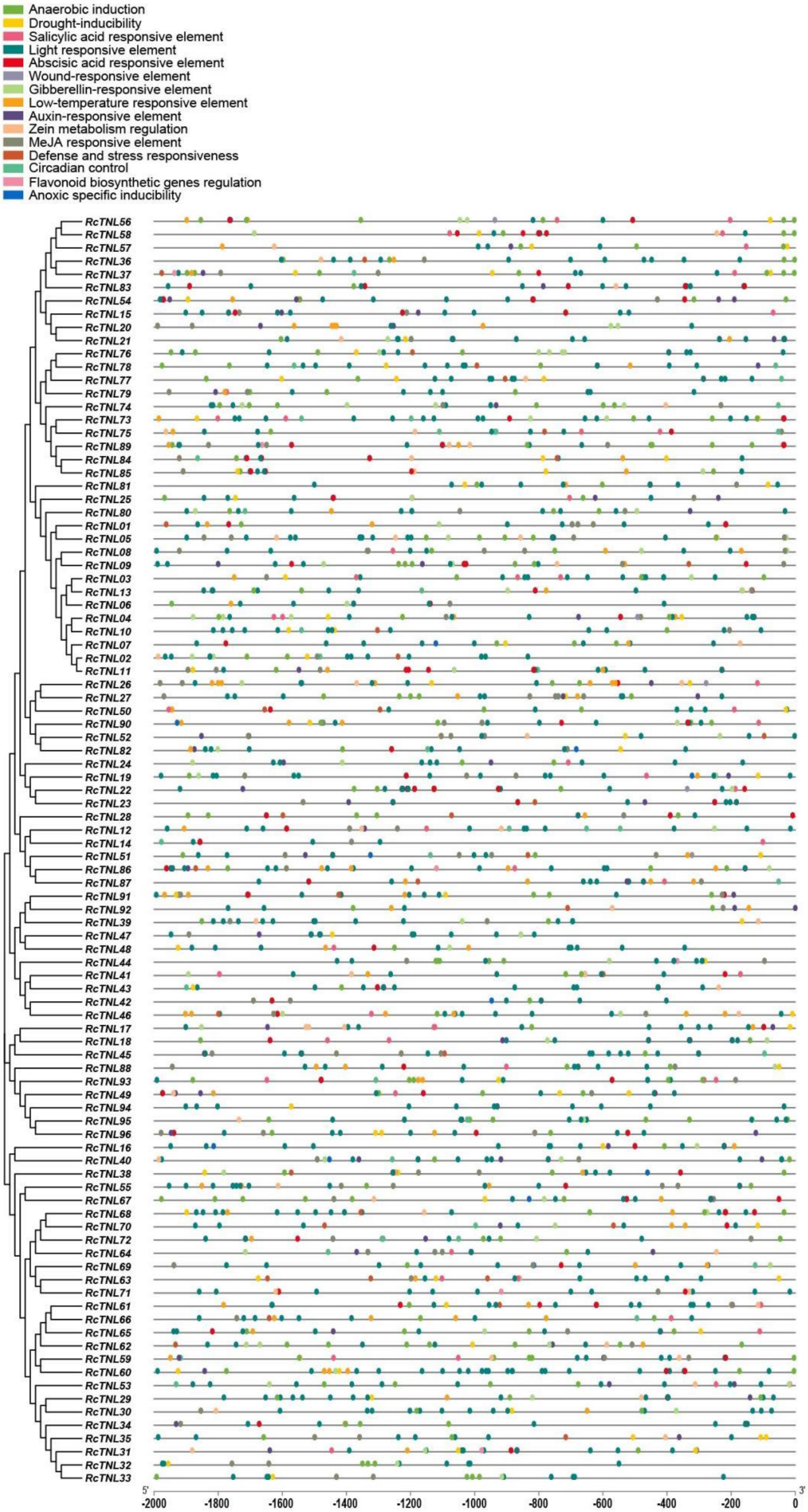

Figure S1. Distribution of cis-elements on the *RcTNL* gene promoters. On the left is the phylogenetic tree of *RcTNL* genes using the maximum likelihood method by MEGA X. The 15 cis-elements are represented by different colors. The right side of the bottom ruler is close to the start codon of gene. The ruler unit is base pair (bp).

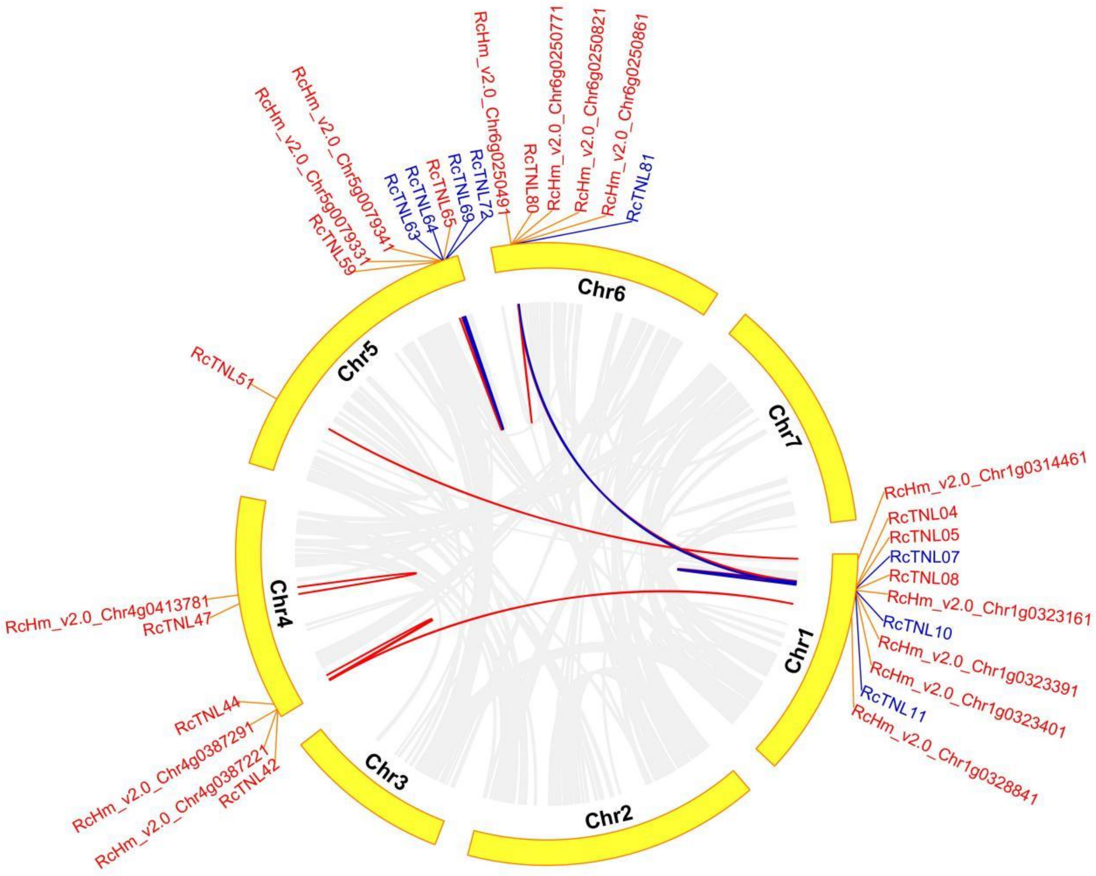

Figure S2. Segmental duplicated *RcTNL* gene pairs in *Rosa chinensis*. The blue, red, and gray lines in the innermost circle indicate segmental duplication events for *RcTNL* and *RcTNL*, *RcTNL* and non-*RcTNL*, and non-*RcTNL* and non-*RcTNL*, respectively. The chromosome number is written in black. The outermost blue and red letters indicate the gene names or gene IDs, of *RcTNL* and *RcTNL*, *RcTNL*, and non-*RcTNL* gene pairs with segmental duplication events, respectively.

a

The internal transcribed spacer (ITS) sequence of DBE24-1:
CGGAAGGATCATTACCGTAGACCCAGCCTGTCGCCGGTCGGCGGCCGGCTGCCTCTGTACCCGTGCCTACCGTACCCCTG
TTGCTTTGGCGAGGCGCCCCTCCGCGGGGCCAGCGGCTCCGGCGGCTGCGTCCTCGCCAGAGACCCGAAAACCCATGCC
TATCAGTGATGTCTGAGCACTATCGAAATAGTTCAAAACTTTCAACAACGGATCTCTTGGTTCTGGCATCGATGAAGAACG
CAGCGAAATGCGATAAGTAATGTGAATTGCAGAATTCAGTGAATCATCGAATCTTTGAACGCACATTGCGCCCTTTGGTAT
TCCGAAGGGCATGCCTGTTCGAGCGTCATTACAACCACTCCAGCCCCTTGCGGCTGGGCCTTGGGGTGCCCGGCTCCGGG
ACCCCTAAAATCAGTGGCGGCGCTGGCCGGCCCTGCGCGCAGTAGACCATCCTCGCGTCTGGGTCCGGCTGGTGTCCTGC
CAGCAACCCCCCCTTTCTCTCCAGGTTGACCTCGGATCAGGTAGGGATACCCGCTGAACTTAAGCATATCA

b

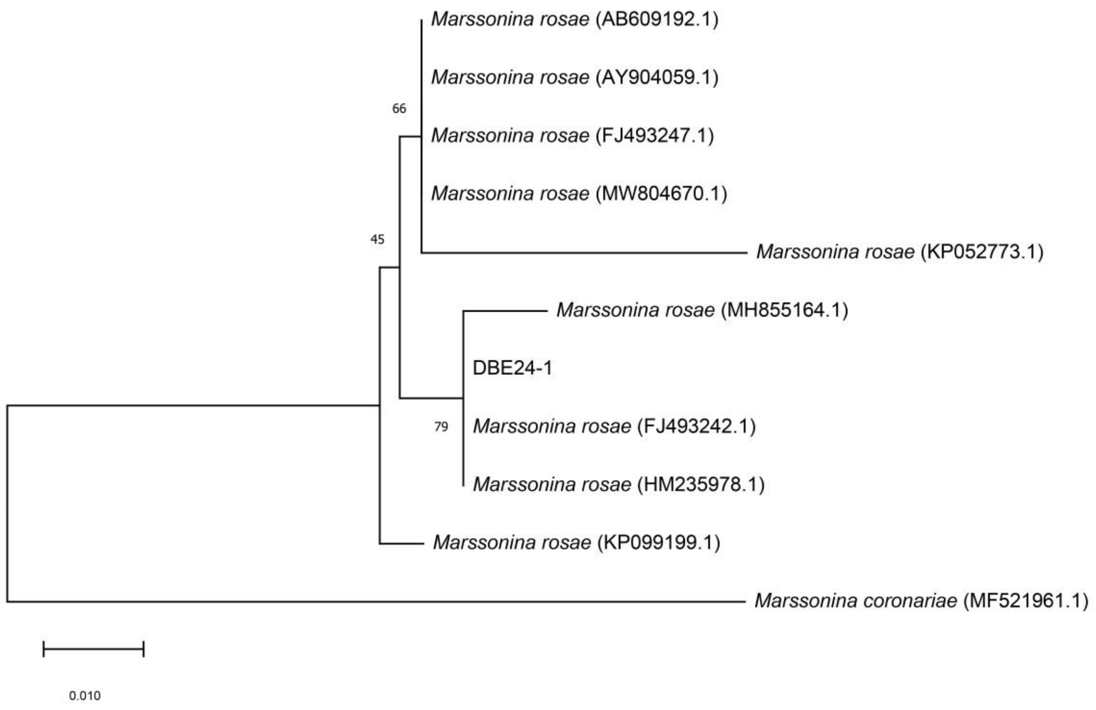

Figure S3. Molecular identification of the black spot pathogen strain DBE24-1 in the rose. (**a**) The internal transcribed spacers (ITS) sequence of DBE24-1 that was amplified with ITS1 and ITS4 primers. (**b**) The phylogenetic tree of DBE24-1 was obtained using ITS sequences via MEGA X. The distance of evolution is represented as the length of branches, and the number to the left of branches indicates the bootstrap values.

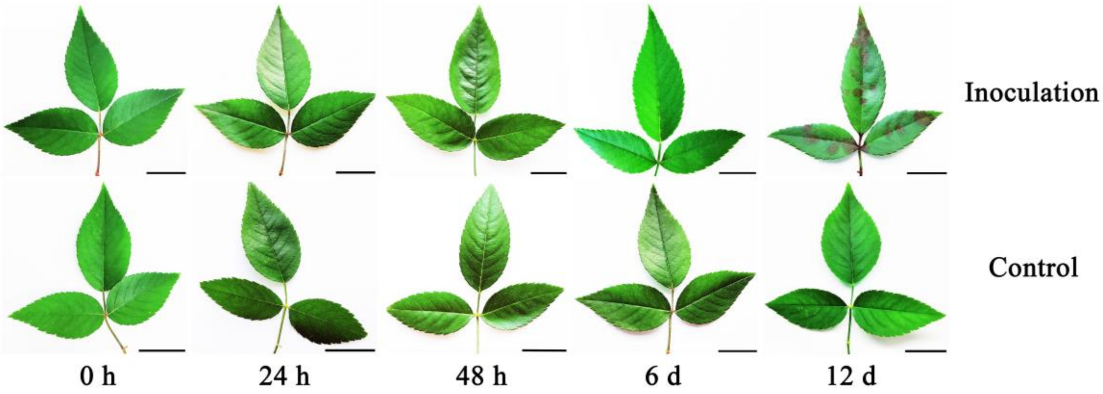

Figure S4. Black spot symptoms on 'Old Blush' leaves after inoculation with the DBE24-1 strain. The upper and lower rows of leaves were inoculated with DBE24-1 conidia (inoculation) and sterile water (control), respectively. Leaves are representations of the symptoms present at 0 h, 24 h, 48 h, 6 d, and 12 d. Bar = 2 cm.
